# Ensuring adequate health financing to prevent and control the COVID-19 in Iran

**DOI:** 10.1186/s12939-020-01181-9

**Published:** 2020-05-06

**Authors:** Masoud Behzadifar, Mahboubeh Khaton Ghanbari, Ahad Bakhtiari, Meysam Behzadifar, Nicola Luigi Bragazzi

**Affiliations:** 1 Social Determinants of Health Research Center, Lorestan University of Medical Sciences, Khorramabad, Iran; 2 Health Management and Economics Research Center, Iran University of Medical Sciences, Tehran, Iran; 3 Department of Health Management and Economics, School of Public Health, Tehran University of Medical Sciences, Tehran, Iran; 4 Department of Epidemiology and Biostatistics, School of Public Health and Nutrition, Lorestan University of Medical Sciences, Khorramabad, Iran; 5 Department of Health Sciences (DISSAL), Postgraduate School of Public Health, University of Genoa, Genoa, Italy; 6 Laboratory for Industrial and Applied Mathematics (LIAM), Department of Mathematics and Statistics, York University, Toronto, ON Canada

**Keywords:** COVID-19, Iran, Pandemics, Health financing, equity

## Abstract

On February 19^th^ 2020, the Iranian Ministry of Health and Medical Education (MoHME) has announced the first 2 cases of SARS-CoV-2, a novel emerging coronavirus which causes an infection termed as COVID-19, in Qom city. As such, the Iranian government, through the establishment of the “National Headquarters for the management and control of the novel Coronavirus”, has started implementing policies and programs for the prevention and control of the virus. These measures include schools and universities closure, reduced working hours, and increased production and delivery of equipment such as masks, gloves and hygienic materials for sterile environments. The government has also made efforts to divulge high-quality information concerning the COVID-19 and to provide laboratories and hospitals with diagnostic kits and adequate resources to treat patients. However, despite such efforts, the number of cases and deaths has progressively increased with rising trends in total confirmed cases and deaths, as well as in new daily cases and deaths associated with the COVID-19. Iran is a developing country and its economic infrastructure has been hit hardly by embargo and sanctions. While developed countries have allocated appropriate funding and are responding adequately to the COVID-19 pandemics, Iran has experienced a serious surge of cases and deaths and should strive to provide additional resources to the health system to make healthcare services more accessible and to increase the fairness of that access. All relevant actors and stakeholders should work together to fight this disease.

## Background

Since late December 2019, a novel coronavirus (SARS-CoV-2), causing a generally mild but sometimes life-threatening infection (termed as COVID-19) has quickly spread out from the epicenter, Wuhan (People’s Republic of China), to other countries [1]. On February 19^th^ 2020, the Iranian Ministry of Health and Medical Education (MoHME) has announced the first 2 COVID-19 cases in Qom city, and the Iranian government, through the establishment of the “National Headquarters for the management and control of the novel Coronavirus”, has started implementing policies and programs for the prevention and control of the virus [2]. These measures include schools and universities closure, reduced working hours, and increased production and delivery of equipment such as masks, gloves and hygienic materials for sterile environments. The government has also made efforts to divulge high-quality information concerning the COVID-19 and to provide laboratories and hospitals with diagnostic kits and adequate resources to treat patients. However, despite such efforts, the number of cases and deaths has progressively increased [2, 3].

Since the beginning of the outbreak, health policy- and decision-makers began to implement intervention policies to prevent, control and treat people in the community. The government has started injecting new funding, being aware of the risks that the disease could pose to the health and other sectors [3]. As a first step, the government allocated 5 billion Tomans to the National Headquarters for the management and control of the outbreak and the country’s medical universities to counteract the burden of the COVID-19 and strengthen the healthcare system. This funding was employed to buy masks, disinfectants and related equipment. Given the population of about 13 million people in Tehran, the capital of Iran and the population density in this metropolitan area, 5 billion Tomans were allocated to equip hospitals and provide essential supplies and rewards to healthcare providers. Also, in the third step, further 5 billion Tomans were allocated to better manage disease control in Qom and Gilan provinces where initially the number of patients was high. Also, the MoHME provided 5 billion Tomans to compensate for physicians, nurses and health personnel in controlling the outbreak [2, 4].

To prevent and control the COVID-19, Iranian policy- and decision-makers decided to quarantine people. Many people were not able to go to work. For this reason, especially poor people experienced serious troubles buying food and other necessities [3]. Therefore, the government decided to provide monthly financial assistance to poor people. Another set of policies was the allocation of 100 billion Tomans and insurance subsidies to the MoHME to prevent the spread of the coronavirus [4].

The Supreme Council for Health and Food Security, along with a special counsel for the COVID-19, has put some essential policies on the agenda to support food security of vulnerable groups, including delays in charging bills such as electricity, repayment of bank loan payments, and giving small amounts of money to vulnerable groups. It should be noted that the government’s capacity to provide such a support has declined due to significant reductions in oil prices and decreased oil selling due to sanctions [3].

In Iran, health policy- and decision-makers are trying to use new financial resources to provide all services. The health system is also providing favourable or free conditions for people who cannot afford to pay for services. With the allocation of new funds, insurers play a major role in protecting patients [5]. Facilitating access to services for many people is a way for further promoting and enhancing justice and equity in health in Iran [6]. However, due to Iran’s sanctions, many health sector activities that require financial exchanges and transactions are in many cases not possible [7]. Many countries around the world, the United Nations and the World Health Organization (WHO) have called for the lifting of sanctions to better deal with the disease [8]. Despite all these problems, the government in Iran has been working to provide new funding to address the COVID-19 [2, 4].

Governmental funding in Iran is mainly dependent on oil revenues, which have declined with the increasing prevalence rate of the COVID-19 and the slump in global production, and oil prices have fallen to their lowest level in the last decades [3, 8, 9]. This is equivalent to reducing income and creating new costs due to the burden of disease. One of the most important financing policies in different countries is to implement programs that do not deprive people of access to health services due to increased out-of-pocket (OOP) payments. Financing the health system is a very important key to ensuring equity in the delivery of health services [10]. However, the COVID-19 pandemics is having a huge impact on different parts of the country, which should strive to ensure flexibility in financing the health system and compensating for the increased costs of disease-related services [11]. A very positive point in the Iranian health system is the attention paid in recent years to the issue of health. The launch of the HTP, the increase of new funding to this sector, especially in the light of the COVID-19 crisis, indicate that the implementation of health policies requires serious government support, commitment and prioritization of this crisis [5].

Iran, as other developing countries, has to face shortage of healthcare personnel, especially nurses [12–14]. The presence of a nurse in wards such as the intensive care unit (ICU) can be of great help in better managing COVID-19 patients. Doctors, nurses and other staff are at the frontline and are working around the clock to help improve patients’ health and reduce mortality. However, unfortunately, a number of dedicated doctors have lost their lives [15].

In order to cope with the shortage of manpower in the health system, the Iranian government increased the number of students that could be admitted in various fields of medical sciences. The government also allowed MoHME to employ 10,000 people to help improve health services, especially in deprived areas [16].

Before the launch of the Health Transformation Plan (HTP) in 2014, there were about 5000 ICU beds. Following the implementation of the HTP, with the allocation of funds to the health sector, beds have been increased by 42% up to 8000, with this increasing trend continuing during the COVID-19 crisis, even though the number of beds is still far from an ideal situation (about 15,000 ICU beds would be needed). Also, the overall number of hospital beds has significantly increased by 25% from 108,000 to more than 130,000, with about 50,000 old beds being renovated. Another major challenge of the Iranian health system in the past was the shortage of ambulances, with 30 air ambulances bases being recently established in Iran. Hospitals have been equipped also with CT, which is crucial for diagnosing the COVID-19 and clinical records have been computerized. To perform an active follow-up in urban and rural areas and to ensure that poor people are guaranteed access, the MoHME has established an electronic health record system during the HTP. Data from this electronic system were combined with data from the Civil Registry Office as well as economic data. Families in various geographical areas were tracked by telephone, and their family’s symptoms were checked. People with current or future early symptoms were called to access to selected health centers; in poor areas, people were granted access to diagnostic and treatment services and were given the addresses of centers where they could be examined. These health centers are part of the country’s health network, which has been developed in rural areas since long time, whereas it has been established in some urban areas (especially those marginalized) in recent years [17, 18].

To better cope with its challenges, the Iran’s health sector has made efforts to improve decision-making processes and the implementation of health policies, with the MoHME as the most important actor coordinating the other sections.

Moreover, in the last four decades sanctions have impacted on Iran’s health sector, limiting the purchase of safety equipment, masks, gloves, clothes for doctors and nurses, as well as medicines, ventilators and diagnostic testing kits. Despite efforts by the WHO and other international organisms to limit these sanctions, embargo policies are still affecting the health sector in Iran.

## Conclusion

Iran is a developing country and its economic infrastructure has been hit hardly by embargo and sanctions. While developed countries have allocated appropriate funding and are responding adequately to the COVID-19 pandemics, Iran has experienced a serious surge of cases and deaths and should strive to provide additional resources to the health system to make services more accessible and to increase the fairness of that access. All relevant actors and stakeholders must work together to fight this disease.

## Data Availability

Not applicable.

## Abbreviations

MoHME: Ministry of Health and Medical Education
HTP: Health Transformation Plan
WHO: World Health Organization
